# Association of Body Mass Index and Waist Circumference with All-Cause Mortality in Hemodialysis Patients

**DOI:** 10.3390/jcm9051289

**Published:** 2020-04-29

**Authors:** Chang Seong Kim, Kyung-Do Han, Hong Sang Choi, Eun Hui Bae, Seong Kwon Ma, Soo Wan Kim

**Affiliations:** 1 Departments of Internal Medicine, Chonnam National University Medical School, Gwangju 61469, Korea; laminion@hanmail.net (C.S.K.); hongsang38@hanmail.net (H.S.C.); baedak@hanmail.net (E.H.B.); drmsk@hanmail.net (S.K.M.); 2 Department of Statistics and Actuarial Science, Soongsil University, Seoul 06978, Korea; hkd917@naver.com

**Keywords:** obesity, body mass index, waist circumference, mortality, hemodialysis

## Abstract

In this study based on a large nationally representative sample of Korean adults, we investigated the potential associations of the body mass index (BMI) and waist circumference (WC) with mortality in patients undergoing hemodialysis. We obtained the data of 18,699 participants >20 years of age who were followed up with for 4 years and for whom BMI and WC information were available, using a nationally representative dataset from the Korean National Health Insurance System. Patients were stratified into five levels by their baseline BMI and into six levels by their WC (5-cm increments). A total of 4975 deaths occurred during a median follow-up period of 48.2 months. Participants with a higher BMI had a lower mortality rate than those with a lower BMI. In a fully adjusted Cox regression analysis, being overweight and obese was associated with a significantly lower relative risk of all-cause mortality relative to the reference group. Conversely, the mortality rate was higher among participants with a high WC than among those with a low WC. Participants with the highest WC had a higher risk of mortality, while those with the lowest WC level had a significantly lower risk of mortality. In conclusion, all-cause mortality was positively associated with WC, a measure of abdominal obesity, and inversely associated with BMI, a measure of body volume, in patients undergoing hemodialysis.

## 1. Introduction

Increases in the prevalence of obesity during the past half-century have led to worldwide health concerns. Obesity particularly increases the risks of several other diseases, including cardiovascular diseases, diabetes, cancer, and chronic kidney disease (CKD) [1,2]. Moreover, obese people tend to face an increased risk of mortality relative to the general population [1]. 

Generally, the body mass index (BMI), an internationally accepted standard anthropomorphic measurement, is used to define obesity in research settings [3]. However, the association of BMI with mortality tends to be discordant in patients with renal impairment, and therefore this population exhibits a so-called “obesity paradox”. Specifically, although a high BMI is associated with all-cause mortality and decreased renal function in patients with earlier stages of CKD, this association is attenuated in patients with advanced CKD [4,5]. Moreover, a few small cohort studies and systemic reviews have reported a link between a low BMI and the risk of mortality in patients undergoing hemodialysis [6,7,8]. Despite these observations, no large population-based cohort study has investigated the spectrum of the obesity paradox with respect to mortality in patients with end-stage renal disease (ESRD). 

Previously, measures of central or abdominal obesity, which is defined by a high waist circumference (WC) and waist-hip ratio, have also been used as predictors of cardiovascular and all-cause mortality [9]. Multiple studies of patients with ESRD have identified the WC as a direct and strong predictor of mortality and incident cardiovascular events, even after adjusting for BMI and other risk factors [10,11]. However, those earlier studies were based mainly on data from Western hemodialysis populations. Therefore, in this study, we examined the relationships of the BMI and WC with the risk of all-cause mortality in a sample of hemodialysis patients included in the Korean National Health Insurance Service (NHIS) database. 

## 2. Materials and Methods

### 2.1. NHIS Data Source

The data used in this study were obtained from a national health insurance claims database established by the Korean NHIS. This database includes all claims data provided by the NHIS and Medical Aid programs. The Korean NHIS is a compulsory social insurance scheme that covers approximately 97% of the Korean population; the remaining 3% are covered by the Medical Aid program [12]. Therefore, data extracted from the NHIS database are considered to represent the entire South Korean population (approximately 50 million residents). All insured Koreans older than 40 years of age undergo a biannual health checkup supported by the NHIS, and employed Koreans older than 20 years are required to undergo an annual health checkup. Body weight (kg), height (cm), WC (cm), systolic blood pressure (mmHg), and diastolic blood pressure (mmHg) are measured during these checkups. In this study, we used a subset of NHIS health checkup data corresponding to the period from 2009 to 2015 [13].

The study protocol was approved by the Institutional Review Board (IRB) of Chonnam National University Hospital (CNUH-EXP-2020-009) and the NHIS (NHIS-2018-1-109). The patients’ identification numbers were anonymized to protect individual privacy. Because only anonymized and de-identified information was used for analysis, the IRB waived the requirement for informed consent. 

### 2.2. Study Population 

**Figure 1:**
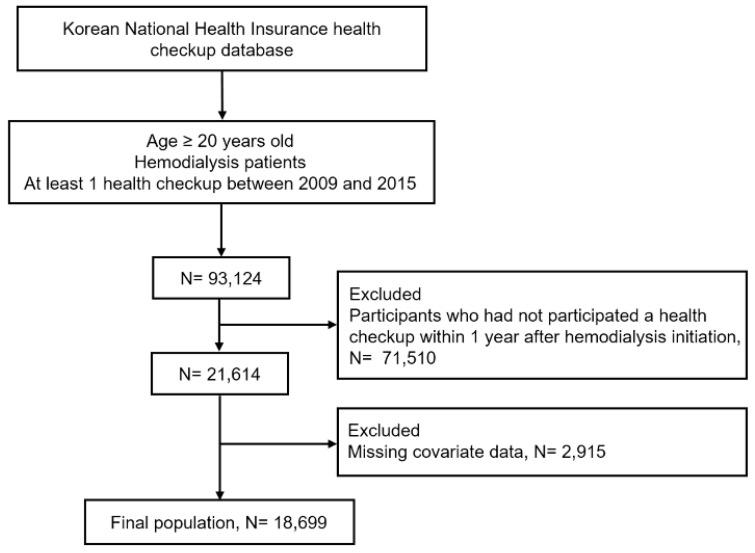
Flow chart of the study population.

This study included the data of 93,124 hemodialysis patients older than 20 years who underwent at least 1 health examination between 2009 and 2015. Hemodialysis patients were identified using a combination of the International Statistical Classification of Diseases and Related Health Problems, 10th Revision (ICD-10) codes (N18–19, Z49, Z94.0 and Z99.2) and a special code (V001, procedure-related outpatient care or inpatient treatment on the day of hemodialysis). The Korean Health Insurance Review and Assessment Service database can be used for the reimbursement of medical expenses for dialysis. Moreover, dialysis patients are registered as special Medical Aid program beneficiaries. Therefore, we were able to use this combination of information to identify every hemodialysis patient in the South Korean population for inclusion in our study. After excluding participants who had not participated in a health checkup within 1 year after hemodialysis initiation (*n* = 71,510) and those with missing covariate data (*n* = 2915), the study population finally comprised 18,699 subjects. A flowchart of the study participant selection process is shown in Figure 1.

### 2.3. Definitions of BMI and WC 

For each participant, the BMI was calculated by dividing the weight (in kg) by the square of the height (in m^2^). We defined obesity as a BMI ≥ 25 kg/m^2^. The participants were then classified into the following categories according to the World Health Organization recommendations for Asian populations: underweight (BMI < 18.5 kg/m^2^), normal (≥18.5 to <23 kg/ m^2^), overweight (≥23 to <25 kg/m^2^), stage 1 obesity (≥25 to 30 kg/m^2^), or stage 2 obesity (≥30 kg/m^2^) [14]. 

The WC of each participant was also measured at the midpoint between the rib cage and the iliac crest by a trained examiner. The patients were divided into 6 categories based on 5-cm WC increments: <80 cm in men and <75 cm in women, 80–85 cm in men and 75–80 cm in women, 85–90 cm in men and 80–85 cm in women (reference group), 90–95 cm in men and 85–90 cm in women, 95–100 cm in men and 90–95 cm in women, and ≥100 cm in men and ≥95 cm in women. Abdominal obesity was defined as WC ≥90 cm in men and ≥85 cm in women according to the definition of the Korean Society for the Study of Obesity [15]. 

### 2.4. Definitions of Health-Behavior Factors and Laboratory Measurements 

The analysis included comorbidities, such as hypertension, diabetes, dyslipidemia, cardiovascular disease, and cancer; and health behavioral factors, such as physical activity, alcohol consumption, and smoking, which are known to be associated with mortality. Hypertension was defined as a previous hypertension diagnosis (ICD-10 codes I10–13, I15) and a history of taking at least 1 antihypertensive drug, or a recorded systolic blood pressure of ≥140 mmHg or diastolic blood pressure of ≥90 mmHg in the health examination database. Diabetes was defined as a previous clinical diagnosis (ICD-10 codes E11–14) and a medical history of diabetes, or a recorded fasting serum glucose concentration of ≥126 mg/dL in the health examination database. Dyslipidemia was identified using the appropriate diagnostic code (E78) and a history of lipid-lowering drug use, or a total serum cholesterol concentration of ≥240 mg/dL in the health examination database. Chronic kidney disease (CKD) was defined as an estimated glomerular filtration rate (eGFR) of <60 mL/min/1.73 m^2^ and was calculated using the Modification of Diet in Renal Disease formula [16]. Cardiovascular disease was identified by the presence of diagnostic codes corresponding to myocardial infarction and stroke (I21–22, I60–69). Cancer was defined as the inclusion of an appropriate diagnostic code (C00–96). The participants’ fasting blood glucose (mg/dL), total cholesterol (mg/dL), triglyceride (mg/dL), high-density lipoprotein cholesterol (mg/dL), and low-density lipoprotein cholesterol (mg/dL) concentrations were measured in a fasting state [13]. The quality of the laboratory tests has been warranted by the Korean Association for Laboratory Medicine, and the hospitals participating in the NHI health checkup programs have been certified by the NHIS.

Participants were additionally categorized into 3 groups according to their smoking status (non-smokers, former smokers, or current smokers) and into 3 categories according to alcohol consumption (none, moderate, or heavy drinkers (≥30 g of alcohol/day)). Participation in regular exercise was defined by the response to the following question: “Did you exercise moderately for >30 min until you were substantially short of breath on more than 5 days during the last week?” Residence in an urban area was also determined.

Finally, the participants were divided into the income quartiles (Q) of Q1 (lowest), Q2, Q3, and Q4 (highest) to assess the effects of socio-economic status. A low income was defined as classification into Q1 or the receipt of Medical Aid benefits. 

### 2.5. Study Outcomes and Follow-Up 

For all participants, the incidence of all-cause mortality between January 1, 2009 and December 31, 2017, was evaluated, and the total number of person-years of follow-up was calculated. A total of 4975 deaths occurred during a median follow-up period of 48.2 months.

### 2.6. Statistical Analyses

The data are presented as numbers or proportions for categorical variables and as means ± standard deviations for continuous variables. Non-normally distributed variables are presented as geometric means (25th, 75th percentiles). To compare the characteristics of interest between cohorts, Student’s t-test was applied to continuous variables, and the Chi-square test was used to assess binary and categorical variables. The mortality rate was calculated per 100 person-years. 

The cumulative incidence probability of all-cause mortality was estimated using the Kaplan–Meier method, and between-group comparisons of the resulting curves were subjected to a univariate analysis via the log-rank test. Multivariable Cox proportional hazard models were developed to determine the hazard ratios (HRs) and 95% confidence intervals (CIs) of the associations between the 5 BMI categories and 6 WC categories with all-cause mortality. In model 1, these calculations were adjusted for age and sex. Model 2 was additionally adjusted for smoking, alcohol consumption, regular exercise, a low income, and previous histories of diabetes, hypertension, dyslipidemia, cardiovascular disease, and cancer. Model 3 included all covariates from model 2 plus the BMI or WC, according to the independent variable, with the intent to reduce the confounding effects of either latter variable. Smooth HR curves of the BMI or WC were plotted after adjusting for all covariates. Finally, the adjusted Cox model (model 2) was used to examine the associations of overweight (BMI ≥23 kg/m^2^) and obesity (BMI ≥25 kg/m^2^) or abdominal obesity (WC ≥90 cm in men and ≥85 cm in women) with all-cause mortality with respect to the 5 BMI groups and 6 WC groups, respectively. 

All statistical tests were two-tailed. A *P* value <0.05 was considered to indicate statistical significance. All analyses were conducted using SAS software (version 9.4; SAS Institute Inc., Cary, NC, USA).

## 3. Results 

### 3.1. Baseline Characteristics 

Table 1 and Table S1 (Supplementary Materials) present the characteristics of participants classified into the five BMI levels and six WC levels. The mean baseline age was 59.9 years, and 56.5% of the sample were men. In our analysis, the WC increased and lipid profile measurements (total cholesterol, triglyceride, low-density lipoprotein and high-density lipoprotein) worsened as the BMI level increased. Moreover, higher BMI categories were associated with an increasing prevalence of diabetes, dyslipidemia, and cardiovascular disease, as well as a greater frequency of alcohol consumption. The proportions of female subjects, subjects with cancer, a low income level or non-regular exercise habits, and non-smokers increased among hemodialysis patients with a low and high BMI, thus yielding U-shaped curves for these associations. 

We similarly evaluated the associations of the WC with various factors. Notably, an increase in the WC was associated with a higher BMI, worse a lipid profile, and a higher prevalence of diabetes, dyslipidemia, and cardiovascular disease. The associations of WC with the proportions of female subjects, subjects with cancer, a low income, or non-regular exercise habits, and non-smokers also yielded U-shaped curves.

### 3.2. Associations of BMI and WC with the Risk of Mortality

**Figure 2:**
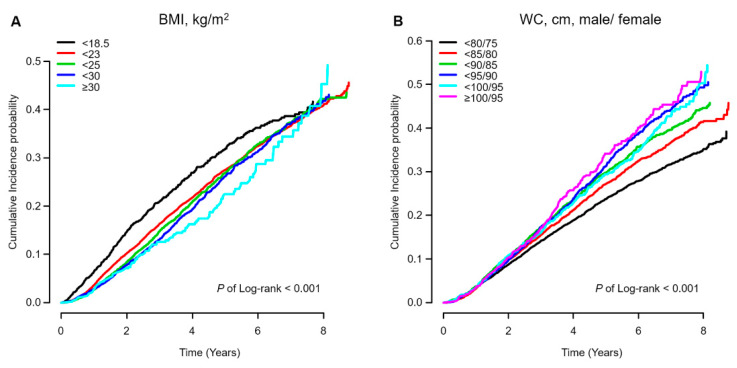
Kaplan–Meier estimation of the cumulative incidence probability of all-cause mortality. The analyses are stratified by (**A**) 5 baseline BMI categories and (**B**) 6 baseline WC categories. BMI, body mass index; WC, waist circumference.

The Kaplan–Meier estimations of the incidence probability of all-cause mortality according to the BMI or WC category are shown in Figure 2. Notably, higher mortality rates were observed at both lower BMI levels and higher WC levels. Table 2 presents the mortality rates and adjusted HRs for all-cause mortality. A total of 4975 deaths occurred during the study period, and the median follow-up time to death or censoring was 48.2 months. After adjusting for all covariates and the WC in model 3, the HR for all-cause mortality decreased as the BMI level increased, with adjusted HRs of 1.67 (95% CI, 1.49–1.87), 0.76 (95% CI, 0.70−0.82), 0.65 (95% CI, 0.59−0.72), and 0.60 (95% CI, 0.48−0.75) for the underweight, overweight, obese, and severely obese groups, respectively, relative to the reference group (normal weight). In summary, a higher BMI was associated with lower all-cause mortality in patients undergoing maintenance hemodialysis.

**Figure 3:**
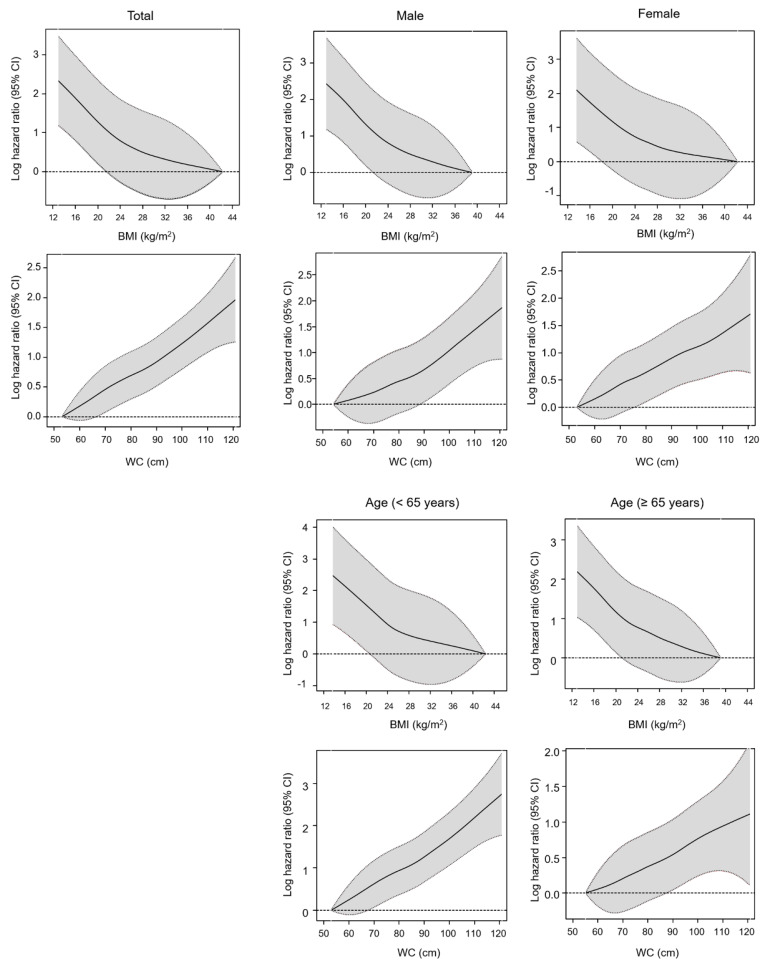
Smoothed hazard ratios curves of the associations of BMI and WC with all-cause mortality in hemodialysis patients, stratified by age and sex. Log hazard ratios are adjusted for age; smoking status; alcohol consumption; regular exercise status; low income; previous history of diabetes, hypertension, dyslipidemia, cardiovascular disease and cancer; and BMI or WC. BMI, body mass index; WC, waist circumference; CI, confidence interval.

Conversely, the adjusted HRs for all-cause mortality increased linearly as the WC increased. In the BMI-adjusted Cox model (model 3), participants with the highest WC values had a higher risk of all-cause mortality than those in the reference group, while those in the lowest WC group had a significantly lower risk of all-cause mortality than those in the reference group (adjusted HRs, 1.68; 95% CI, 1.45−1.96 and HR, 0.80; 95% CI, 0.73−0.88, respectively). These associations were confirmed by smooth HR curve analyses performed after the adjustment of all covariates (Figure 3). Specifically, a negative linear association was observed between the BMI and risk of all-cause mortality, whereas a positive linear association was observed between the WC and the risk of all-cause mortality. We observed the same associations between BMI and WC and all-cause mortality in an age and sex-stratified subgroup analysis. However, the U-shaped association of the WC with all-cause mortality was observed among subgroups who had diabetes, cardiovascular disease, or cancer, suggesting that the body weight loss or malnutrition also had a high association with all-cause mortality as well as abdominal obesity in patients with these comorbidities that affected the body weight and WC (Figure S1, Supplementary Materials). 

### 3.3. Subgroup Analyses of the Association of Obesity and Abdominal Obesity with All-Cause Mortality According to the BMI or WC Level

**Figure 4:**
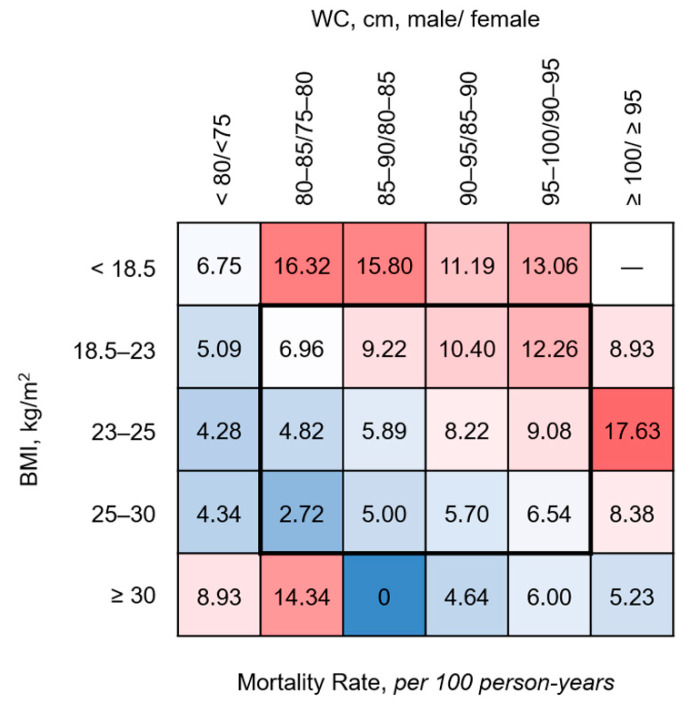
**Mortality rates according to the 5 baseline BMI categories and 6 baseline WC categories.** Each square indicates the morality rate calculated per 100 person-years for the variable indicated by the row and column. Red and blue colors indicate higher and lower mortality rates, respectively. BMI, body mass index; WC, waist circumference.

We further explored the associations between all-cause mortality and obesity and abdominal obesity after stratifying patients into the five BMI categories and six WC categories. Notably, an increased mortality rate was observed among patients with a lower BMI and higher WC, whereas a decreased mortality rate was observed in the higher BMI and lower WC groups. However, the highest or lowest BMI and WC groups were excluded from these trends (Figure 4).

**Figure 5:**
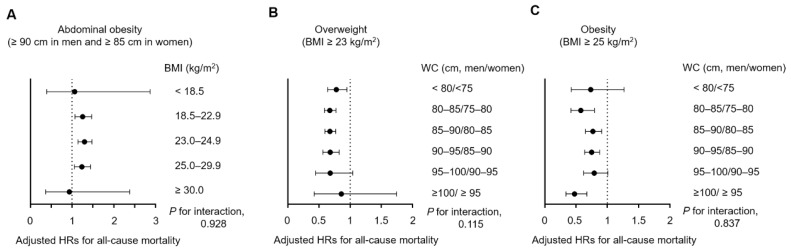
Subgroup analyses of the associations of (**A**) abdominal obesity, (**B**) overweight, and (**C**) obesity with all-cause mortality according to the BMI or WC group (as appropriate). BMI, body mass index; HR, hazard ratio; WC, waist circumference.

Next, we evaluated the association between abdominal obesity, as defined by the WC, and all-cause mortality in the five BMI subgroups to investigate the adverse effect of this variable when modified by BMI (Figure 5A). In the normal weight, overweight, and stage 1 obesity subgroups, abdominal obesity was associated with a significant increase in the adjusted HR for all-cause mortality (relative to non-abdominal obesity). However, the statistically significant association between abdominal obesity and all-cause mortality disappeared in the underweight and stage 2 obesity subgroups. We found no evidence of interaction among the BMI subgroups (*P* for the interaction, 0.928). 

We also analyzed the association of overweight and obesity, as defined by the BMI, with all-cause mortality in the six WC subgroups. Conversely, we observed lower adjusted HRs for all-cause mortality in patients with an overweight or obese BMI across all WC subgroups (Figure 5B,C). There were no statistically significant interactions among the WC subgroups. 

## 4. Discussion 

In our large Korean population-based study of patients undergoing hemodialysis, we identified associations of a low BMI and a 5-cm increase in WC with an increased risk of all-cause mortality. These observed linear associations of the BMI and WC with mortality remained significant, even after adjusting for the WC or BMI, respectively. Moreover, abdominal obesity was associated with an increased risk of mortality (relative to non-abdominal obesity) among normal weight or obese patients receiving hemodialysis. Conversely, an obese BMI was associated with a lower risk of mortality, regardless of the WC. These associations persisted even after adjusting for multiple confounding variables, including smoking status, alcohol consumption, exercise habits, and various comorbidities. 

Previous studies reported associations of obesity or abdominal obesity with mortality and decreased renal function in both the general population [1,13,17,18,19,20] and among patients with CKD [1,4,5,21,22,23,24]. Most studies based on general populations and patients with early-stage CKD indicated positive associations of the BMI or WC with the risks of a low eGFR and mortality [1,13,17,18,19,20]. However, a recent meta-analysis of a cohort of CKD patients revealed a relatively weaker association between a higher BMI and low eGFR [1]. Similarly, a large cohort study of veterans in the United States also detected an attenuated association between a high BMI and mortality in patients with advanced CKD [5], and two meta-analyses reported an inverse association between the BMI and mortality in patients with stage 3–5 CKD who were not receiving dialysis [4,22]. These observations further support the obesity paradox.

Several factors and mechanisms have been proposed to explain the inverse relationship between BMI and all-cause mortality in patients with CKD. For example, Ladhani et al., suggested that obese patients with CKD have survived the traditional factors associated with obesity, such as hypertension, diabetes mellitus, and cardiovascular disease [22]. Moreover, obesity may be associated with a more stable hemodynamic status and better nutritional reserves, and stronger protective cytokine profiles [25] might also confer a relative survival advantage upon obese individuals [21,26,27]. In line with previous studies of patients with CKD, a recent meta-analysis of patients receiving hemodialysis reported that a 1-kg/m^2^ increase in the BMI was associated with a 5% decrease in the risk of all-cause mortality [7]. We similarly observed a strong inverse relationship between the BMI and the risk of all-cause mortality even after adjusting for the WC, as demonstrated by a 40% decrease in the mortality risk faced by severely obese patients (BMI ≥30 kg/m^2^) relative to that of normal weight patients receiving hemodialysis. Our findings are consistent with recently published data from a Korean hemodialysis registry, which reported an association of overweight (BMI ≥30 kg/m^2^) with a reduced risk of mortality in patients older than 40 years [28]. 

Recent studies have suggested that metabolically unhealthy obesity and a visceral pattern of body fat deposition [3,29] are more important contributors to a risk assessment of mortality than the overall “body volume” as measured by the BMI. The WC, a representative marker of visceral body fat, was found to correlate with inflammation, whereas subcutaneous body fat may be an indicator of the nutritional status [30]. In previous studies, measures of fat distribution and abdominal obesity that are based on the WC or waist-hip ratio have been found to associate directly with mortality in both general populations [1,13,18] and in cohorts of patients with CKD [23,31,32] and ESRD [8,10,11,33,34,35,36]. In the Reasons for Geographic and Racial Differences in Stroke (REGARDS) study, patients with CKD who presented abdominal obesity (≥122 cm in men and ≥108 cm in women) had a 2-fold higher risk of mortality than their counterparts without abdominal obesity [23]. In a study of the Calabria Registry of Dialysis and Transplantation (CREDIT) registry, which is based in Europe, a 10-cm increase in the WC remained associated with a 26% increase in the risk of mortality and 38% increase in the risk of cardiovascular mortality, even after adjusting for the BMI; in contrast, the probability of death appeared to be minimal among patients with a high BMI and low WC [10]. Furthermore, the CREDIT study reported associations of abnormally high concentrations of triglycerides, cholesterol, or adipose tissue cytokines (e.g., adiponectin and leptin) with increased all-cause and cardiovascular mortality, especially in patients with abdominal obesity who were undergoing hemodialysis [11,35]. Several studies with smaller populations also indicated that abdominal obesity, defined by the WC, was the single most significant predictor of cardiovascular events in patients receiving hemodialysis [33,36] and of mortality in patients receiving peritoneal dialysis [34]. Similar to those previous studies, our study also observed an association of an increased WC with all-cause mortality in Korean patients receiving hemodialysis, even after adjusting for the BMI. Particularly, patients in the highest WC category in our study had a 68% higher HR for mortality relative to the reference group. 

Our results suggest that patients with a low BMI and high WC should have the highest risk of mortality, as observed in the CREDIT study [10]. However, our subgroup analyses stratified by BMI did not find that lean hemodialysis patients with abdominal obesity had the highest risk of all-cause mortality (Figure 4 and Figure 5). In other words, we did not observe that abdominal obesity was associated with a higher risk of mortality in our subgroup of underweight patients. This discrepancy between studies may be attributable to the importance of malnutrition as a predictor of mortality in patients receiving hemodialysis. Specifically, the extreme increase in the HR of mortality among underweight patients may have outweighed the adverse effects of abdominal obesity. We therefore assume that severely underweight patients who require hemodialysis face an increased risk of all-cause mortality, regardless of their abdominal obesity status.

This study has several limitations. First, we were unable to determine the causes of death and variables related to comorbidities (e.g., pulmonary or liver disease), the type of vascular access and dialysis technique, inflammatory markers, and nutrition factors due to the limitations of the database [37,38,39,40,41]. Nevertheless, we attempted to adjust the models to include health-behavior factors and comorbidities (e.g., cardiovascular disease and cancer) that might have affected the body weight and WC data and mortality. Second, the adjusted HRs were based on BMI or WC values measured at a single time point, and we did not consider changes in these values over time. Recently, however, a large study that adjusted for the confounding effect of changing values over time reported similar, robust conclusions when using conventional models and marginal structural model analyses [42]. Third, the duration of follow-up from the collection of anthropomorphic measurements until death (or censoring) was short to enable the evaluation of long-term mortality. However, our study provided a relatively long-term evaluation compared to that reported in most previous studies, which had a follow-up of <4 years [10,33,34,36]. Finally, this study population only comprised patients receiving hemodialysis who had undergone a health examination. This may have led to selection bias, as the enrolled final population might have been somewhat healthier than the excluded participants. However, all patients undergoing hemodialysis visit hemodialysis centers, where they are subjected to monthly laboratory examinations; therefore, these patients tend to avoid participating in further health examinations, regardless of their health status. Our study also had some notable strengths. As noted above, most previous related studies involved Western populations. Therefore, ours is the first study of the relationships of BMI and WC with the risk of all-cause mortality in a large population of Korean patients undergoing hemodialysis who were included in a well-established and validated longitudinal national database. Moreover, the WC measurements of all participants were determined and recorded by trained examiners at certified facilities. 

In conclusion, our findings indicate that abdominal obesity, measured according to the WC, is associated with all-cause mortality in Korean patients undergoing hemodialysis. In contrast, body volume, as measured by BMI, exhibited an inverse relationship with all-cause mortality. Our results suggest that the routine measurement of both BMI and WC would provide useful information about the risk of mortality faced by patients undergoing hemodialysis. Although no Korean guideline currently provides recommendations for obesity management in patients undergoing hemodialysis, we suggest that a therapeutic intervention intended to preserve muscle mass while reducing abdominal body fat would ultimately improve patients’ outcomes.

## Figures and Tables

**Table 1:** Baseline characteristics of the study population by body mass index.

Characteristics	Total	BMI, kg/m^2^					
		<18.5	18.5–23	23–25	25–30	≥30	*P* Value
Number	18,699	1264	9534	3985	3439	477	
Age, years, mean ± SD	59.9 ± 10.9	57.0 ± 13.0	59.5 ± 11.0	61.2 ± 10.3	60.8 ± 10.3	56.8 ± 11.2	<0.001
Age group, years (%)							<0.001
20–40	565 (3.0)	92 (7.3)	303 (3.2)	78 (2.0)	65 (1.9)	27 (5.7)	
40–65	11,697 (62.6)	807 (63.8)	6066 (63.6)	2396 (60.1)	2107 (61.3)	321 (67.3)	
≥ 65	6437 (34.4)	365 (28.9)	3165 (33.2)	1511 (37.9)	1267 (36.8)	129 (27.0)	
Female (%)	8128 (43.5)	760 (60.1)	4077 (42.8)	1516 (38.0)	1498 (43.6)	277 (58.1)	<0.001
Place of residence (%)							0.209
Urban living	8360 (44.7)	551 (43.6)	4331 (45.4)	1783 (44.7)	1490 (43.3)	205 (43.0)	
Rural living	10,339 (55.3)	713 (56.4)	5203 (54.6)	2202 (55.3)	1949 (56.7)	272 (57.0)	
Smoking (%)							<0.001
Non	12,410 (66.4)	970 (76.7)	6296 (66.0)	2559 (64.2)	2249 (65.4)	336 (70.4)	
Former	4273 (22.9)	184 (14.6)	2161 (22.7)	1006 (25.2)	834 (24.3)	88 (18.5)	
Current	2016 (10.8)	110 (8.7)	1077 (11.3)	420 (10.5)	356 (10.4)	53 (11.1)	
Alcohol consumption							0.018
None (%)	17,046 (91.2)	1189 (94.1)	8705 (91.3)	3608 (90.5)	3114 (90.6)	430 (90.2)	
Moderate (%)	1535 (8.2)	71 (5.6)	767 (8.0)	353 (8.9)	301 (8.8)	43 (9.0)	
Heavy (%)	118 (0.6)	4 (0.3)	62 (0.7)	24 (0.6)	24 (0.7)	4 (0.8)	
Regular exercise (%)							<0.001
No	16,050 (85.8)	1125 (89.0)	8086 (84.8)	3401 (85.3)	3009 (87.5)	429 (89.9)	
Yes	2649 (14.2)	139 (11.0)	1448 (15.2)	584 (14.7)	430 (12.5)	48 (10.1)	
Diabetes mellitus (%)	8659 (46.3)	450 (35.6)	3999 (41.9)	1975 (49.6)	1927 (56.0)	308 (64.6)	<0.001
Hypertension (%)	14,820 (79.3)	982 (77.7)	7589 (79.6)	3142 (78.9)	2730 (79.4)	377 (79.0)	0.554
Dyslipidemia (%)	6677 (35.7)	322 (25.5)	2972 (31.2)	1546 (38.8)	1560 (45.4)	277 (58.1)	<0.001
CKD (%)	17,989 (96.2)	1216 (96.2)	9178 (96.3)	3827 (96.0)	3312 (96.3)	456 (95.6)	0.911
Cardiovascular disease (%)	4325 (23.1)	268 (21.2)	2096 (22.0)	952 (23.9)	898 (26.11)	111 (23.3)	<0.001
Cancer (%)	555 (3.0)	42 (3.3)	298 (3.1)	103 (2.6)	96 (2.8)	16 (3.4)	<0.001
Low income (%)							<0.001
No	11710 (62.6)	735 (58.2)	5934 (62.2)	2593 (65.1)	2175 (63.3)	273 (57.2)	
Yes	6989 (37.4)	529 (41.8)	3600 (37.8)	1392 (34.9)	1264 (36.7)	204 (42.8)	
BMI, kg/m^2^, mean ± SD	22.7 ± 3.2	17.5 ± 0.9	21.0 ± 1.2	23.9 ± 0.6	26.7 ± 1.3	32.2 ± 2.1	<0.001
WC, cm, mean ± SD	80.7 ± 9.6	68.2 ± 6.4	76.6 ± 6.7	84.0 ± 6.0	90.3 ± 6.8	100.2 ± 8.2	<0.001
SBP, mmHg, mean ± SD	134.3 ± 20.1	132.7 ± 20.6	134.3 ± 20.2	134.4 ± 19.8	134.2 ± 19.9	136.7 ± 19.9	0.005
DBP, mmHg, mean ± SD	77.9 ± 11.8	78.1 ± 12.3	77.9 ± 11.9	77.8 ± 11.5	77.8 ± 11.7	79.8 ± 12.1	0.008
Fasting glucose, mg/dL, mean ± SD	114.5 ± 47.6	109.2 ± 45.9	111.6 ± 46.0	115.5 ± 47.4	120.8 ± 50.7	129.6 ± 53.0	<0.001
Total cholesterol, mg/dL, mean ± SD	166.8 ± 39.1	165.9 ± 37.9	165.8 ± 38.2	167.7 ± 39.7	167.6 ± 40.2	174.4 ± 45.1	<0.001
High-density lipoprotein, mg/dL, mean ± SD	49.0 ± 15.3	55.7 ± 17.1	51.1 ± 15.3	46.8 ± 14.9	44.2 ± 13.3	43.4 ± 11.9	<0.001
Low-density lipoprotein, mg/dL, mean ± SD	93.3 ± 32.8	90.0 ± 30.7	92.9 ± 32.0	94.7 ± 33.7	93.9 ± 33.9	95.0 ± 37.3	<0.001
Triglyceride, mg/dL, (25th 75th)	108.0 (107.2–108.8)	91.2 (89.0–93.4)	97.8 (96.9–98.7)	116.3 (114.5–118.1)	131.6 (129.4–133.7)	160.2 (152.6–168.1)	<0.001

Abbreviations: CKD, chronic kidney disease; BMI, body mass index; WC, waist circumference; SBP, systolic blood pressure; DBP, diastolic blood pressure; SD, standard deviation.

**Table 2:** Mortality rates and hazard ratios of all-cause mortality by BMI or WC.

Group	Number	Death	Follow-Up Duration, Person-Year	Mortality Rate, Per 100 Person-Year	Model 1, HR (95% CI) ^a^	Model 2, HR (95% CI) ^b^	Model 3, HR (95% CI) ^c^
BMI group, kg/m^2^							
<18.5	1264	380	5146.1	7.38	1.46 (1.31–1.62)	1.48 (1.33–1.65)	1.67 (1.49–1.87)
18.5–23	9534	2586	41213.6	6.27	reference	reference	reference
23–25	3985	1059	17444.3	6.07	0.87 (0.81–0.93)	0.84 (0.78–0.90)	0.76 (0.70–0.82)
25–30	3439	846	14609.4	5.79	0.86 (0.80–0.93)	0.80 (0.74–0.86)	0.65 (0.59–0.72)
≥30	477	104	1987.5	5.23	1.03 (0.85–1.25)	0.86 (0.70–1.04)	0.60 (0.48–0.75)
WC group, cm, (male/female)							
<80/<75	7006	1640	31042.7	5.28	1.00 (0.92,1.08)	1.08 (1.00–1.18)	0.80 (0.73–0.88)
80–85/75–80	4125	1100	17857.8	6.16	0.97 (0.89,1.06)	0.99 (0.91–1.08)	0.88 (0.80–0.96)
85–90/80–85	3368	968	14165.0	6.83	reference	reference	reference
90–95/85–90	2167	657	8990.5	7.31	1.02 (0.93–1.13)	1.00 (0.90–1.10)	1.12 (1.01–1.24)
95–100/90–95	1140	338	4789.0	7.06	1.06 (0.94–1.20)	0.97 (0.86–1.10)	1.25 (1.10–1.42)
≥100/≥95	893	272	3555.8	7.65	1.19 (1.04–1.36)	1.07 (0.93–1.22)	1.68 (1.45–1.96)

^a^ Model 1, adjusted for age and sex ^b^ Model 2, adjusted for age, sex, smoking, alcohol drinking, regular exercise, low income, and previous history of diabetes, hypertension, dyslipidemia, cardiovascular disease, and cancer. ^c^ Model 3, Adjusted for age, sex, smoking, alcohol drinking, regular exercise, low income, previous history of diabetes, hypertension, dyslipidemia, cardiovascular disease, cancer, and BMI or WC. Abbreviations: HR, hazard ratio; CI, confidential interval; BMI, body mass index; WC, waist circumference.

## Supplementary Materials

The following are available online at https://www.mdpi.com/2077-0383/9/5/1289/s1, Table S1: Baseline characteristics of the study population by and waist circumference, Figure S1: Smoothed hazard ratios curves of the associations of BMI and WC with all-cause mortality in hemodialysis patients, stratified by diabetes, hypertension, cardiovascular disease and cancer.
