# Supplementary material for: Association of Body Mass Index and Waist Circumference with All-Cause Mortality in Hemodialysis Patients
Source: J Clin Med. 2020 Apr 29;9(5):1289. doi: 10.3390/jcm9051289 (PMC7288310; doi:10.3390/jcm9051289)

Table S1. Baseline characteristics of the study population by and waist circumference.

Characteristics	WC, male/ female, cm						
	< 80/<75	80–85/75–80	85–90/80–85	90–95/85–90	95–100/90–95	≥ 100/ ≥ 95	*P* value
Number	7006	4125	3368	2167	1140	893	
Age, years, mean ± SD	57.3±11.5	60.4±10.5	61.9±10.0	62.5±10.0	61.7±10.3	61.5±10.5	< 0.001
Age group, years (%)							< 0.001
20–40	343(4.9)	105(2.6)	46(1.4)	31(1.4)	25(2.2)	15(1.7)	
40–65	4779(68.2)	2584(62.6)	1960(58.2)	1202(55.5)	653(57.3)	519(58.1)	
≥ 65	1884(26.9)	1436(34.8)	1362(40.4)	934(43.1)	462(40.5)	359(40.2)	
Female (%)	3276(46.8)	1534(37.2)	1363(40.5)	922(42.6)	538(47.2)	495(55.4)	< 0.001
Place of residence (%)							0.625
Urban living	3134(44.7)	1876(45.5)	1511(44.9)	965(44.5)	489(42.9)	385(43.1)	
Rural living							
Smoking (%)							< 0.001
Non	4783(68.3)	2596(62.9)	2209(65.6)	1446(66.7)	747(65.5)	629(70.4)	
Former	1456(20.8)	1051(25.5)	806(23.9)	512(23.6)	271(23.8)	177(19.8)	
Current	767(11.0)	478(11.6)	353(10.5)	209(9.6)	122(10.7)	87(9.7)	
Alcohol consumption							0.046
None (%)	6406(91.4)	3737(90.6)	3085(91.6)	1955(90.2)	1031(90.4)	832(93.2)	
Moderate (%)	562(8.1)	352(8.5)	271(8.1)	195(9.0)	100(8.8)	55(6.2)	
Heavy (%)	38(0.5)	36(0.9)	12(0.4)	17(0.8)	9(0.8)	6(0.7)	
Regular exercise (%)							< 0.001
No	5962(85.1)	3487(84.5)	2879(85.5)	1876(86.6)	1033(90.6)	813(91.0)	
Yes	1044(14.9)	638(15.5)	489(14.5)	291(13.4)	107(9.4)	80(9.0)	
Diabetes mellitus (%)	2477(35.4)	1910(46.3)	1753(52.1)	1207(55.7)	686(60.2)	626(70.1)	< 0.001
Hypertension (%)	5494(78.4)	3325(80.6)	2693(80.0)	1708(78.8)	894(78.4)	706(79.1)	0.097
Dyslipidemia (%)	1982(28.3)	1494(36.2)	1292(38.4)	907(41.9)	524(46.0)	478(53.5)	< 0.001
CKD (%)	6726(96.0)	3966(96.2)	3226(95.8)	2101(97.0)	1109(97.3)	861(96.4)	0.091
Cardiovascular disease (%)	1282(18.3)	1024(24.8)	846(25.1)	589(27.2)	332(29.1)	252(28.2)	<0.001
Cancer (%)	220(3.1)	121(2.9)	88(2.6)	64(3.0)	35(3.1)	27(3.0)	<0.001
Low income (%)							< 0.001
No	4205(60.0)	2615(63.4)	2201(65.4)	1413(65.2)	732(64.2)	544(60.9)	
Yes	2801(40.0)	1510(36.6)	1167(34.7)	754(34.8)	408(35.8)	349(39.1)	
BMI, kg/m^2, mean ± SD	20.3±1.9	22.4±1.8	23.7±2.0	25.0±2.2	26.6±2.4	29.2±3.3	< 0.001
WC, cm, mean ± SD	71.5±5.0	80.1±2.8	84.8±2.8	89.6±2.8	94.3±2.9	101.7±5.0	< 0.001
SBP, mmHg, mean ± SD	133.6±20.3	135.0±20.1	134.1±19.7	134.2±19.9	134.7±20.1	135.8±20.7	0.002
DBP, mmHg, mean ± SD	78.2±12.0	78.1±11.6	77.6±11.5	77.2±11.9	77.4±11.8	78.0±11.8	0.005
Fasting glucose, mg/dL, mean ± SD	107.2±43.3	114.3±45.8	117.5±49.0	120.8±51.3	123.4±51.9	133.6±55.5	< 0.001
Total cholesterol, mg/dL, mean ± SD	166.8±37.9	165.0±38.4	167.6±40.0	167.6±40.0	166.5±40.7	170.3±42.9	0.002
High-density lipoprotein, mg/dL, mean ± SD	53.5±15.9	48.6±15.2	46.6±13.8	44.4±12.9	43.6±13.3	42.4±12.8	< 0.001
Low-density lipoprotein, mg/dL, mean ± SD	92.6±31.7	92.6±32.4	95.0±33.4	94.6±33.9	93.0±33.9	93.8±36.1	0.004
Triglyceride, mg/dL, (25th 75th)	92.8(91.8–93.8)	106.0(104.4–107.5)	116.4(114.5–118.3)	127.1(124.5–129.7)	132.6(128.8–136.5)	151.6(146.5–156.9)	<0.001

Abbreviations: CKD, chronic kidney disease; BMI, body mass index; WC, waist circumference; SBP, systolic blood pressure; DBP, diastolic blood pressure; SD, standard deviation.

Figure S1. Smoothed hazard ratios curves of the associations of BMI and WC with all-cause mortality in hemodialysis patients, stratified by diabetes, hypertension, cardiovascular disease and cancer. Log hazard ratios are adjusted for age; sex; smoking status; alcohol consumption; regular exercise status; low income; previous history of diabetes, hypertension, dyslipidemia, cardiovascular disease and cancer; and BMI or WC, appropriately. BMI, body mass index; WC, waist circumference; CI, confidence interval.

Figure S1

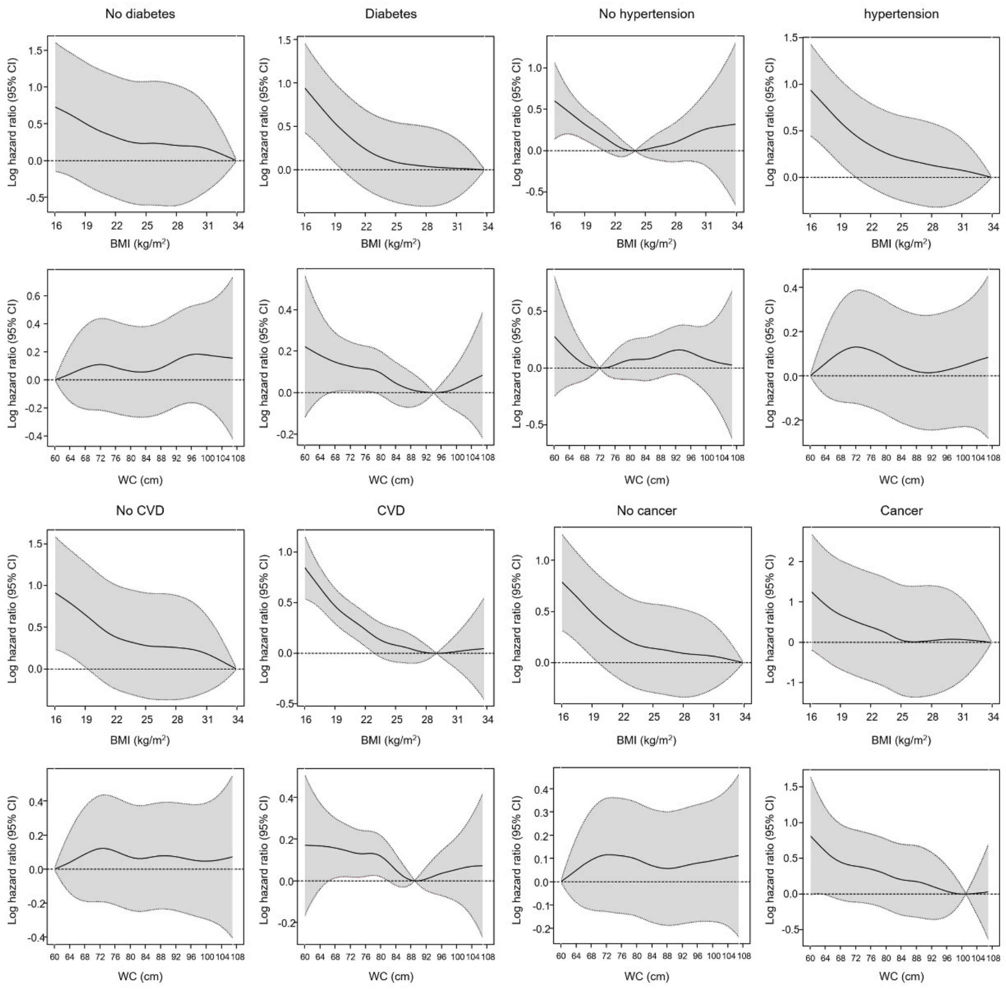

Supplement: Supplementary file 1 [file jcm-09-01289-s001.pdf]
